# Genetic Variation in *VEGF* Does Not Contribute Significantly to the Risk of Congenital Cardiovascular Malformation

**DOI:** 10.1371/journal.pone.0004978

**Published:** 2009-03-24

**Authors:** Helen R. Griffin, Darroch H. Hall, Ana Topf, James Eden, A. Graham Stuart, Jonathan Parsons, Ian Peart, John E. Deanfield, John O'Sullivan, Sonya V. Babu-Narayan, Michael A. Gatzoulis, Frances A. Bu'Lock, Shoumo Bhattacharya, Jamie Bentham, Martin Farrall, Javier Granados Riveron, J. David Brook, John Burn, Heather J. Cordell, Judith A. Goodship, Bernard Keavney

**Affiliations:** 1 Institute of Human Genetics, Newcastle University, Newcastle upon Tyne, United Kingdom; 2 Congenital Heart Centre, Bristol Royal Hospital for Children, Bristol, United Kingdom; 3 Paediatric Cardiology Unit, Leeds General Infirmary, Leeds, United Kingdom; 4 Department of Paediatric Cardiology, Alder Hey Royal Children's Hospital, Liverpool, United Kingdom; 5 Cardiothoracic Unit, Great Ormond Street Hospital, London, United Kingdom; 6 Congenital Heart Unit, Cardiothoracic Centre, Freeman Hospital, Newcastle upon Tyne, United Kingdom; 7 Royal Brompton Hospital and National Heart & Lung Institute, Imperial College, London, United Kingdom; 8 Congenital and Paediatric Cardiology Service, Glenfield Hospital, Leicester, United Kingdom; 9 Department of Cardiovascular Medicine, University of Oxford, Wellcome Trust Centre for Human Genetics, Oxford, United Kingdom; 10 Institute of Genetics, University of Nottingham, Queen's Medical Centre, Nottingham, United Kingdom; Leiden University Medical Center, Netherlands

## Abstract

Several previous studies have investigated the role of common promoter variants in the vascular endothelial growth factor (*VEGF*) gene in causing congenital cardiovascular malformation (CVM). However, results have been discrepant between studies and no study to date has comprehensively characterised variation throughout the gene. We genotyped 771 CVM cases, of whom 595 had the outflow tract malformation Tetralogy of Fallot (TOF), and carried out TDT and case-control analyses using haplotype-tagging SNPs in *VEGF*. We carried out a meta-analysis of previous case-control or family-based studies that had typed *VEGF* promoter SNPs, which included an additional 570 CVM cases. To identify rare variants potentially causative of CVM, we carried out mutation screening in all *VEGF* exons and splice sites in 93 TOF cases. There was no significant effect of any *VEGF* haplotype-tagging SNP on the risk of CVM in our analyses of 771 probands. When the results of this and all previous studies were combined, there was no significant effect of the *VEGF* promoter SNPs rs699947 (OR 1.05 [95% CI 0.95–1.17]); rs1570360 (OR 1.17 [95% CI 0.99–1.26]); and rs2010963 (OR 1.04 [95% CI 0.93–1.16]) on the risk of CVM in 1341 cases. Mutation screening of 93 TOF cases revealed no *VEGF* coding sequence variants and no changes at splice consensus sequences. Genetic variation in *VEGF* appears to play a small role, if any, in outflow tract CVM susceptibility.

## Introduction

Congenital cardiovascular malformation (CVM) occurs in approximately 7 out of every 1000 live births [1], and is the commonest cause of death in childhood in developed countries. CVM is defined as a gross structural abnormality of the heart or intrathoracic great vessels that is present at birth and is of functional significance. The incidence figure quoted above excludes bicuspid aortic valve, which is present in 1–2% of live births, but is usually not detected until much later in life, if at all. Though some 20% of cases of CVM can be attributed to specific causes such as chromosomal disorders, recognised multi-organ syndromes and teratogen exposure, the majority of cases are assumed to result from a complex interaction of environmental and genetic factors [2], [3]. In support of this notion, substantial genetic influences have been inferred for certain malformations in studies of familial recurrence risk ascertained through non-syndromic patients [4]. However, as yet relatively few candidate genes have been systematically investigated for any possible contribution of common variants to disease risk.

Vascular endothelial growth factor (*VEGF*) plays an important role in the formation of the endocardial cushions during heart development, and either up- or down-regulation of VEGF expression can result in CVM in experimental models [5]–[14]. Genotypes at the common *VEGF* promoter SNPs rs699947 and rs1570360, and the 5′UTR SNP rs2010963 are associated with inter-individual differences in *VEGF* expression levels *in vitro* [15], [16]. Genotypes and haplotypes at these SNPs that are associated with lower VEGF production were first associated with the risk of CVM in a study which compared 58 patients with microdeletion of 22q11 and CVM with 316 healthy controls. This suggested that VEGF could act as a genetic modifier of the cardiac manifestations of 22q11 deletion [12]. The associations were confirmed in a trio-based study of 148 non-syndromic patients with the outflow tract malformation Tetralogy of Fallot (TOF); no association was found in that study among 40 trios containing a proband with transposition of the great arteries [17]. It was therefore suggested that the VEGF haplotypes had a specific association with TOF. TOF is a particularly important CVM sub-phenotype for genetic study since it is the commonest complex cyanotic heart defect (which invariably requires corrective surgery in early life), and familial recurrence risk studies have shown particularly strong evidence for genetic effects. A subsequent study comparing 102 patients with non-syndromic valvular and/or septal defects of the heart and 112 controls found no association between genotype at a proxy SNP for rs699947 and CVM, and found association in the opposite direction to the previous studies between the rs2010963 SNP and CVM [18]. Most recently, the genotypes and haplotypes associated with lower VEGF expression were associated with disease risk in a study of 222 Chinese patients with Ventricular Septal Defect (VSD) and 352 controls [19]. Data from the literature is only available in 570 patients at present, and estimates of the population attributable risk (PAR) of CVM arising from common genetic variation in VEGF range from 0.11 to 0.48 in previous studies (our calculations). More precise estimates of these potentially important genetic risks are therefore required from larger studies and pooled analyses of available data. We have carried out a comprehensive characterisation of common variants in VEGF which more than doubles the number of CVM cases studied thus far. We have combined the results of this study and all previous studies in a meta-analysis. Our primary dataset includes a large number of cases with the outflow tract malformation TOF, which should enable us to address the potential sub-phenotypic specificity of the previously published association securely.

Since CVM has historically been a condition with a high perinatal and childhood mortality, it is evolutionarily plausible that low-frequency, intermediate-penetrance variants could make at least as significant a contribution to the population genetic risk as common variants. However, no previous study has systematically carried out mutation screening of the VEGF gene in CVM cases. We therefore also carried out exonic and splice site resequencing in 93 TOF cases to identify whether individually rare variants in VEGF collectively make a significant contribution to TOF risk.

The hypotheses tested by this study were therefore twofold: first, that common genetic variants in VEGF were associated with the occurrence of non-syndromic, non-Mendelian CVM (in particular, TOF); and second, that rarer variants affecting amino-acid sequence or consensus splice sites were associated with the occurrence of non-syndromic, non-Mendelian TOF.

## Results

The founder genotypes for twenty VEGF Haplotype-tagging SNPs (htSNPs) were shown to be in Hardy-Weinberg equilibrium (p>0.01). Genotype and haplotype frequencies were in good agreement with HapMap data, for those markers where it was available, and with previous published studies. The likelihood ratio statistics and associated p-values from the UNPHASED analysis of the twenty VEGF htSNPs in both TOF and non-TOF CVM families and controls are shown in Table 1. None of the SNPs showed a significant association with CVM in 357 TOF cases. When the three previously reported SNPs (rs699947, rs1570360, rs2010963) were genotyped in an additional 238 TOF cases, there remained no significant association in the total of 595 TOF cases. Similarly, none of the VEGF htSNPs showed significant association with CVM risk in the 176 cases with non-TOF CVM. Although borderline significant (p = 0.035–0.037) evidence for association was observed at rs1547651 and rs3025035 in the non-TOF CVM patients, this seems unlikely to reflect true association when multiple comparisons are taken into account. The three SNPs reported in previous studies to show association with CVM (rs699947, rs1570360, and rs2010963) were also analysed as a three-marker haplotype in the 595 TOF probands and the 176 CVM probands using UNPHASED. There was no association between any of the four haplotypes and either TOF or non-TOF CVM (Table 2).

**Table 1:** Association analysis of *VEGF* genotypes.

SNP	TOF (357)		TOF (595)		CVM (176)	
	LR	p-value	LR	p-value	LR	p-value
rs833052	0.88	0.65			1.30	0.52
rs866236	0.18	0.91			3.72	0.16
rs833057	0.65	0.72			1.93	0.38
rs1547651	0.37	0.83			6.72	0.04
rs833058	0.11	0.95			0.25	0.89
rs699946	0.11	0.95			0.50	0.78
**rs699947**	**2.29**	**0.32**	**1.70**	**0.43**	**0.48**	**0.79**
**rs1570360**	**3.14**	**0.21**	**3.90**	**0.14**	**0.93**	**0.63**
**rs2010963**	**0.36**	**0.84**	**1.29**	**0.53**	**3.64**	**0.16**
rs2146323	0.81	0.67			1.52	0.47
rs3025000	0.07	0.97			3.37	0.19
rs3025033	0.76	0.69			0.11	0.95
rs3025035	0.48	0.79			6.61	0.04
rs9369421	0.32	0.85			3.30	0.19
rs879825	0.07	0.97			1.75	0.42
rs1358980	3.60	0.17			0.84	0.66
rs1885658	0.20	0.91			1.66	0.44
rs1885659	1.43	0.49			1.44	0.49
rs10948095	0.62	0.73			0.39	0.82
rs13210960	0.38	0.83			0.51	0.77

Likelihood Ratio (LR) Chi-Squared statistics with associated probabilities (p-value) for ‘UNPHASED’ analysis of VEGF genotypes in the 357 and 595 TOF families and 176 CVM families; three previously reported SNPs are shown in bold.

**Table 2:** Association analysis of three-marker *VEGF* haplotype.

TOF (595)									
		Haplotype	Case	Control	Ca-Freq	Co-Freq	OR	95%Low	95%High
**LR**	4.11	A-A-G*	389	352	0.33	0.33	1.00	1.00	1.00
**df**	3	A-G-G	181	182	0.15	0.17	0.85	0.67	1.07
**p-value**	0.25	C-G-C†	399	332	0.34	0.31	1.07	0.89	1.29
		C-G-G	205	190	0.17	0.18	0.94	0.76	1.17

CVM (176)									
		Haplotype	Case	Control	Ca-Freq	Co-Freq	OR	95%Low	95%High
**LR**	4.63	A-A-G*	111	189	0.32	0.32	1.00	1.00	1.00
**df**	3	A-G-G	62	104	0.18	0.18	0.85	0.54	1.35
**p-value**	0.20	C-G-C†	101	196	0.29	0.33	0.75	0.51	1.11
		C-G-G	72	100	0.21	0.17	1.16	0.77	1.74

Likelihood Ratio (LR) Chi-Squared statistics with associated probabilities (p-value); estimated allele counts and frequencies in cases and controls (Ca-Freq, Co-Freq); and odds ratios with 95% confidence intervals from ‘UNPHASED’ analysis of VEGF rs699947 (−2578A/C)/ rs1570360 (−1154A/G) / rs2010963 (−634C/G) haplotype in the 595 TOF families and 176 CVM families are presented.

* Haplotype −2578A/−1154A/−634G, previously reported by Lambrechts et al. (2005) and Stalmans et al. (2003) to increase risk of TOF and CVM.

† Haplotype −2578C/−1154G/−634C, previously reported by Xie et al. (2007) to be protective against VSD.

The search strategy identified four previous studies, all of which were suitable for inclusion in the meta-analysis. Genotype frequencies were either stated to, or could be calculated to conform to, Hardy-Weinberg equilibrium in all studies. Genotype frequencies were generally in good agreement between the studies, although the study by Vannay et al. [18] showed a significantly different allele frequency at the rs2010963 SNP in controls from that observed in the other studies or from dbSNP data for subjects of European ancestry. Vannay et al. [18] typed SNP rs833061 (referred to in that study as T-460C) which was not typed in other studies; however, HapMap data from the CEU population indicates that this is an almost perfect proxy SNP for rs699947 (D′ = 1; r^2^ = 0.965) and therefore the rs833061 SNP data from that study was pooled with the data for rs699947 derived from the other studies. The study by Lambrechts et al. [17] contained sub-populations with TOF and transposition of the great arteries; odds ratios were calculated separately for each population for each SNP before pooling. The study by Xie et al. [19] included 222 cases with valvuloseptal defects who were compared with 352 unrelated controls; of the 222 cases in that study 142 had parents available. Xie et al. [19] carried out TDT testing in that subgroup, obtaining slightly more extreme odds ratios (but wider confidence intervals) in the smaller dataset. We carried out pooled analyses including either the case/control comparison or the TDT subgroup from that study; there was no material difference in the conclusions whichever population was included (data available on request). We therefore principally quote the analyses including the larger dataset of 222 cases and 352 unrelated controls from Xie et al. [19]. The pooled analyses contain information on 1341 CVM cases, of whom 743 have TOF, 540 have non-TOF CVM, and 58 have CVM associated with 22q11 deletion. Removal of the 58 patients with CVM and 22q11 deletion did not, as expected given their small numbers, alter any of the conclusions of the pooled analyses.

The pooled analysis for rs699947 yielded a combined OR for CVM of 1.05 (95% CI 0.95–1.17) in all patients, 1.02 (95% CI 0.88–1.18) in patients with TOF, and 1.04 (95% CI 0.88–1.22) in patients with non-TOF CVM for the A allele associated with risk in smaller studies (Table 3). As can be inferred from the 95% CIs, none of these associations were statistically significant at the p<0.05 level. The maximum plausible PAR associated with this SNP for all CVM was calculated as 0.08. The pooled analysis for rs1570360 yielded a combined OR for CVM of 1.12 (95% CI 0.99–1.26) in all patients, 1.09 (95% CI 0.93–1.27) in patients with TOF, and 1.03 (95% CI 0.84–1.27) in patients with non-TOF CVM for the A allele (Table 4). None of these associations were statistically significant, and the maximum plausible PAR associated with this SNP for all CVM was 0.08. The pooled analysis for rs2010963 yielded a combined OR for CVM of 1.04 (95% CI 0.93–1.16) in all patients, 0.99 (95% CI 0.85–1.15) in patients with TOF, and 1.07 (95% CI 0.90–1.26) in patients with non-TOF CVM for the G allele. None of these associations were statistically significant, and the maximum plausible PAR associated with this SNP for all CVM was 0.09 (Table 5).

**Table 3:** Pooled analysis of rs699947: A allele of C/A SNP designated high risk.

Study	No. Probands	OR	95%CI Low	95% CI High
Stalmans	58	1.50	1.00	2.30
Lambrechts TOF	148	1.43	1.03	1.97
Lambrechts TGA	40	1.13	0.57	2.21
Vannay	102	0.79	0.54	1.15
Xie Case-Control (all)	222	1.18	0.89	1.56
Xie TDT only	142	1.08	0.74	1.56
Present study TOF patients	595	0.93	0.79	1.10
Present study other CVM	176	1.04	0.80	1.35
**All studies** *	**1341**	**1.05**	**0.96**	**1.17**

* Pooled ORs include the entire population of cases and controls from Xie et al. (2007) rather than the subset of TDT families.

**Table 4:** Pooled analysis of rs1570360: A allele of G/A SNP designated high risk.

Study	No. Probands	OR	95% CI Low	95% CI High
Stalmans	58	1.80	1.20	2.70
Lambrechts TOF	148	1.46	1.02	2.09
Lambrechts TGA	40	0.94	0.48	1.86
Vannay	not typed			
Xie Case-Control (all)	222	1.10	0.80	1.53
Xie TDT only	142	0.92	0.58	1.46
Present study TOF patients	595	1.02	0.85	1.21
Present study other CVM	176	1.00	0.75	1.33
**All studies** *	**1239**	**1.12**	**0.99**	**1.26**

* Pooled ORs include the entire population of cases and controls from Xie et al. (2007) rather than the subset of TDT families.

**Table 5:** Pooled analysis of rs2010963: G allele of G/C SNP designated high risk.

Study	No. Probands	OR	95% CI Low	95% CI High
Stalmans	58	1.30	0.80	2.00
Lambrechts TOF	148	1.43	1.00	2.02
Lambrechts TGA	40	1.23	0.59	2.56
Vannay	102	0.37	0.24	0.56
Xie Case-Control (all)	222	1.32	1.04	1.68
Xie TDT only	142	1.60	1.16	2.23
Present study TOF patients	595	0.91	0.78	1.08
Present study other CVM	176	1.35	0.97	1.87
**All studies** *	**1341**	**1.04**	**0.93**	**1.16**

* Pooled ORs include the entire population of cases and controls from Xie et al. (2007) rather than the subset of TDT families.

To determine whether low frequency, intermediate penetrance genetic variants in *VEGF* might predispose in particular to Tetralogy of Fallot, the exonic and splice site regions of the gene were re-sequenced in a panel of 93 cases, all of whom had TOF. The two previously reported SNPs rs2010963 and rs25648 were detected in the 5′UTR of exon 1 in a proportion of patients, consistent with previously available data; however, no new coding sequence variants or splice site consensus sequence were identified. Simple binomial probabilities indicate that if such variants were present in as few as 5% of patients in the population with TOF, resequencing of 93 cases would have a greater than 90% power to detect at least one occurrence.

## Discussion

This study does not support the hypothesis that either common or rare genetic variation in VEGF significantly predisposes to the risk of CVM. Our primary data with respect to common variation has more than doubled the amount of information hitherto available in the literature. Pooled analysis of our primary data and previous published studies shows no compelling evidence for association of any of the three previously typed SNPs in the promoter and 5′ region of VEGF with CVM. Furthermore, our analyses show no evidence for a specific effect of VEGF SNPs on the risk of TOF, an important CVM sub-phenotype previously claimed to be particularly strongly associated with the VEGF polymorphisms we have typed. Resequencing of the exonic and splice site regions of *VEGF* in a panel of 93 cases with TOF did not identify any mutations changing amino acids or splice site consensus sequences, suggesting that such mutations could at most account for a very small proportion of the population risk of TOF.

Previous studies had suggested association between VEGF SNPs and CVM; however, numbers of cases in previous studies were small and the confidence intervals around the odds ratio estimates correspondingly large. The data from previous studies suggested that the PAR of SNPs in VEGF was between 0.11 and 0.48, which if the upper estimate were correct would indicate that a very substantial degree of population morbidity and mortality from CVM is directly due to genetic variability in VEGF. However, when our primary data is pooled with the results of previous studies, we show that the maximum plausible PAR from these VEGF SNPs is in fact less than 10%. Very large studies involving several thousand cases would be required to exclude effects of only a few percent on risk. Lambrechts et al. [17] reported an association between the low *VEGF* expression haplotype comprising the A, A, and G alleles at rs699947, rs1570360 and rs2010963 respectively and disease risk in 148 TOF trio families. This study genotyped the same SNPs in a total of 595 TOF probands, 237 of which were trio families, and found no significant over transmission of the low-expression AAG haplotype. The complementary CGC haplotype at these SNPs was reported by Xie et al. [19] to be under-transmitted to 364 probands with VSD. Xie et al. [19] studied a larger number of VSD cases than were present in our primary data, and therefore a protective effect of the CGC haplotype that is specific to VSD cannot be entirely ruled out. The data reported by Vannay et al. [18] was generated in patients with valvuloseptal defects which would very likely have included a high proportion of VSDs; however, no detailed phenotypic breakdown of patients is available in that paper. Moreover, it was not possible to infer the corresponding haplotype frequencies from the data presented in Vannay et al. [18], which precluded a comprehensive haplotype-based meta-analysis of all published data. With respect to our resequencing experiment, no previous similar study has been conducted so far; we resequenced sufficient patients to determine that amino-acid or splice-site altering mutations could at the most account for only a few percent of the population risk of disease.

Animal models have demonstrated that VEGF must be expressed within a physiological window for normal embryonic development, particularly of the heart, to occur [5]–[12], [14]. The mouse Vegf-164 isoform is crucial for cardiac development and a reduction or absence in expression severely perturbs epithelial to mesenchymal transformation and cardiac cushion development [12]. The report of a TOF-like phenotype in mouse models unable to express the Vegf-164 isoform [14] additionally suggests that VEGF should be a strong candidate gene for susceptibility to TOF in humans. Given these considerations, it is perhaps surprising that we demonstrated no association with CVM in this large study, which was adequately powered to detect even small effects on risk. However, it remains possible that severe genetically determined disruptions of VEGF signalling are sufficiently deleterious to heart formation in man that affected embryos are non-viable, in which case such alterations would not be observed in those surviving to birth. We specifically ruled patients with 22q11 deletion out of the present study, therefore we can neither confirm nor refute the assertion of Stalmans et al. [12] that the VEGF promoter haplotype is a modifier influencing the risk of CVM in the setting of 22q11 deletion. It remains possible that among people sharing the 22q11 deletion genotype, there is a deletion-related reduction in genetic “buffering” of the differential activity of the VEGF gene mediated by the promoter variants. If this were so, the VEGF promoter variants might influence CVM susceptibility in persons with 22q11 deletion to a greater extent than they do among those who share the TOF phenotype, but who do not have such a defect in “buffering”. Further studies specifically examining that hypothesis in larger numbers of patients with 22q11 deletion would be of interest.

Despite the large number of samples we investigated, this study has certain limitations. While we have taken care to maximise phenotypic homogeneity among the patients with outflow tract defects that we studied, focusing on TOF and phenotypes that are clinically and developmentally closely related, it is not known for certain whether this group of phenotypes have a common genetic underpinning. It is therefore possible, though we think it unlikely, that heterogeneity of genotype/phenotype relationships among our patients with outflow tract defects reduced the power of this study. Our group of non-outflow tract CVMs was heterogeneous, so if VEGF genotype predisposed to one sub-phenotype within that group (for example, septal defects), it is unlikely that we would have detected it. Our SNP analyses were limited to the VEGF region and 15 Kb either side; therefore, the effects of any long-range regulatory variants not in significant LD with the SNPs we typed might have been missed. Although such variants are well known for several genes, none has yet been described for VEGF to our knowledge.

Hitherto, there are relatively few studies that have examined associations between common SNPs and CVM and, perhaps because of a lack of readily available samples derived from epidemiological studies, most investigators have studied only small numbers of patients. This study shows that, in CVM as in other complex disorders, large sample sizes are of key importance in obtaining reliable results in genetic association studies. Given the relative difficulty in establishing large cohorts for CVM (particularly where particular sub-phenotypes are sought in large numbers), we suggest that collaborative meta-analyses involving multiple cohorts are a useful strategy to provide robust information on candidate genetic susceptibility factors, pending the availability of collaborative genome-wide association study data in large numbers of cases and controls. Despite our negative findings, it remains possible that common and rare *VEGF* genetic susceptibility variants to CVM exist; however, their effect sizes are likely to be small. Much larger patient numbers for genotyping and sequencing would be required to detect such variants, which would be unlikely to have major impacts on the population burden of disease.

## Materials and Methods

### Ethics Statement

Ethical approval was given for the study by the Northern and Yorkshire Multicentre Research Ethics Committee. Fully informed written consent was obtained from all participants (or their parents, if they were children too young to themselves consent). The research was conducted in accordance with the Helsinki declaration.

### Study population

Patients with CVM were recruited from collaborating UK paediatric cardiology centres. Those with clinical diagnoses of del22q11 syndrome or other known chromosomal abnormalities were excluded, as were those with other multi-organ malformation syndromes, learning difficulties, or known maternal exposure to significant teratogens during pregnancy. Specifically, patients with tetralogy of Fallot (TOF: EPCC Code 01.01.01), or with the related conditions pulmonary stenosis/VSD (EPCC Codes 01.01.06 and 01.01.25), and “Fallot-type” double outlet right ventricle (EPCC Code 01.01.17), who were of European ancestry, were recruited together with their parents where available as part of the CHANGE (Congenital Hearts – A National Gene-Environment Study) programme. Samples from 357 TOF patients (202 case-parent trios, 81 case-parent duos, 68 single probands and 3 multiplex families) were genotyped for all selected VEGF SNPs. An additional 238 TOF probands and available parents (35 Trios, 17 duos, 186 single probands), ascertained using the same criteria from centres in Nottingham and Oxford were genotyped at the three previously reported SNPs and rs1547651. Patients of European ancestry affected by other types of CVM were recruited in Newcastle. A total of 176 families (50 trios, 66 duos, 60 single probands) with non-TOF CVM were genotyped (see Table S1 for case phenotypes). These represented the maximal numbers of families available for this study for SNP analysis, and enabled us to more than double the number of cases genotyped in the literature thus far. Controls were selected from a collection of healthy British Caucasian families, ascertained through a proband with essential hypertension, that have been previously described [20]. No control individual had a history of CVM. A total of 182 unrelated control individuals were genotyped and included in the association analysis. A panel of 93 TOF probands from the centres in Bristol, Leeds and Newcastle were selected for exonic re-sequencing of *VEGF*. This number of probands would provide 85% power to detect at least one mutation that affected the amino acid sequence or splice sites, if such mutations were present in 2% of TOF cases or greater. DNA was extracted from blood or saliva samples using standard protocols.

### SNP genotyping

**Figure 1:**
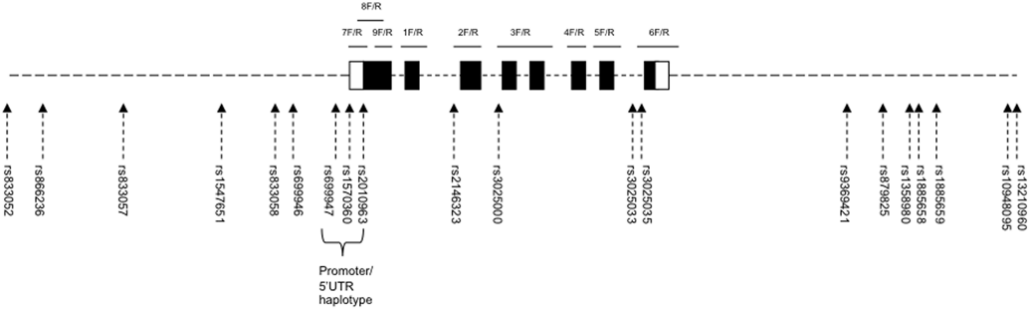
Schematic diagram of VEGF based on transcript NM_003376.3; exons are represented as boxes (shaded – coding, unshaded - UTR), introns, up- and down-stream regions as dotted lines; SNPs were selected 15 Kb upstream, within and 15 Kb downstream of VEGF; the location of SNPs is indicated by dashed arrows; the three SNPs in the previously reported haplotype are bracketed; the approximate position of PCR products for sequencing are represented as solid lines with forward (F) and reverse (R) primer pairs indicated; the proportions of exons, introns, up- and down-stream regions are not to scale.

htSNPs were selected for genotyping from within and 15 KB up- and down-stream of *VEGF* using the HapMap data for the samples of Northern and Western European ancestry (CEU samples: www.broad.mit.edu/mpg/haploview) and the Tagger utility of Haploview v3.2. Eighteen SNPs with MAF>0.05 were selected that were in linkage disequilibrium at r^2^>0.8 with all other genetic variation within the region. The SNP rs699947 previously reported to be associated with TOF was among the selected htSNPs. The other two SNPs typed in previous studies, rs1570360 and rs2010963, which were not genotyped in the HapMap, were additionally selected for genotyping. The location of the SNPs is shown in figure 1.

Seventeen SNPs were genotyped within four iPLEX MALDI-TOF assays (Sequenom). iPLEX PCR and extension primer sequences are shown in Table S2. Multiplex PCRs were performed in 10 µl reactions comprising 1 U Hotstar Taq (Qiagen), with 3.5 mM MgCl_2_, 0.5 mM dNTPs (Invitrogen), 0.1 µM of each primer (Metabion) and 20 ng DNA. Thermal cycling conditions were 95°C for 15 minutes followed by 35 cycles of 95°C for 20 seconds, 56°C for 30 seconds, 72°C for 1 minute and a final extension of 72°C for 3 minutes. PCR products were SAP treated (Sequenom) and iPLEX extension reactions (Sequenom) were performed according to manufacturer's protocols. Three SNPs (rs1570360, rs699946 & rs866236) were genotyped using Taqman pre-designed allelic discrimination assays (Applied Biosystems).

### Exon Resequencing

PCR primers were designed within the introns of *VEGF* based on transcript NM_003376.3; see Table S3 for PCR primer sequences and figure 1 for the location of the primers. PCR was performed in 10 µl reactions comprising 0.25 U Hotstar Taq (Qiagen), 1.5 mM MgCl_2_, 1× Q Solution (Qiagen), 0.8 mM dNTPs (Invitrogen), 0.5 µM of each PCR primer (Metabion) and 20 ng of DNA. Thermal cycling conditions were 95°C for 15 minutes followed by 35 cycles of 95°C for 45 seconds, 55.5°C for 45 seconds, 72°C for 1 minute and a final extension at 72°C for 10 minutes. PCR products were cleaned with Exo-SAP-IT (Amersham) and sequencing reactions performed using MegaBACE DYEnamic ET dye terminator kit (Amersham) following manufacturer's protocols. Sequencing products underwent isopropanol precipitation and were separated using the MegaBace 1000 (Amersham). The Staden Package suite of programs (http://staden.sourceforge.net/) was used to analyse the sequence traces.

### Statistical Analysis

Genotype data was checked for Mendelian consistency within the families and Hardy-Weinberg equilibrium using PEDSTATS [21]. Association analysis of the individual SNPs, and of the previously reported three-SNP haplotype in the VEGF promoter was performed using UNPHASED version 3.0.9 [22]. UNPHASED permits the analysis of datasets containing, as here, both family-based and case-control data, producing a summary statistic for all included individuals.

Previous studies that had investigated the relationship between polymorphisms of VEGF and CVM were sought by Medline searches using a variety of search string combinations (e.g. “VEGF” and “congenital heart”; “VEGF” and “tetralogy of Fallot”), and by hand-searching of reference lists in studies so identified. Studies utilising either a case-control or a family-based design were included. For an identified study to be eligible for inclusion, sufficient information had to be available for the numbers of cases and controls with each genotype to be deducible (for case-control studies), or for the numbers of transmitted and untransmitted risk alleles from heterozygote parents to affected offspring to be deducible (for studies in family trios). Odds ratios and 95% confidence intervals were calculated for each SNP in each study using standard formulae, assuming Hardy-Weinberg equilibrium and a multiplicative penetrance model [23]. Studies were combined to produce summary odds ratios and 95% confidence intervals using an inverse-variance weighting approach. We prespecified subgroup analyses of the combined data in patients with TOF and patients with other forms of CVM, in view of previous published data suggesting a specific effect of VEGF genotypes on TOF. Since it was not possible to deduce the three-SNP VEGF promoter haplotype frequencies in all the identified studies, meta-analysis for the haplotype was not conducted. We calculated the plausible upper limit of the population attributable risk (PAR) due to each SNP by substituting the upper 95% CI of the pooled OR estimate and the frequency of the risk allele (F) into the equation PAR = F (OR-1)/(F(OR-1)+1).

## Supporting Information

Table S1 (0.09 MB DOC)

Table S2 (0.06 MB DOC)

Table S3 (0.04 MB DOC)
