# Supplementary material for: Genetic Variation in VEGF Does Not Contribute Significantly to the Risk of Congenital Cardiovascular Malformation
Source: PLoS One. 2009 Mar 24;4(3):e4978. doi: 10.1371/journal.pone.0004978 (PMC2654913; doi:10.1371/journal.pone.0004978)
Supplement: Table S1 — (0.09 MB DOC) [file pone.0004978.s001.doc]

**Table S1 Phenotypes of 176 CVM patients**

| **Septal Malformations** | **No. Cases** |
| --- | --- |
| **VSD** | **17** |
| with Pulmonary stenosis | 3 |
| with ASD | 3 |
| with PDA | 2 |
| with hypoplastic right ventricle | 2 |
| with aortic regurgitation | 2 |
| tricuspid atresia | 1 |
| IAA | 1 |
| with left ventricular outflow obstruction | 1 |
| **ASD** | **43** |
| with pulmonary stenosis | 2 |
| with aneurismal intra-atrial septum | 2 |
| with tricuspid & mitral regurgitation | 2 |
| with VSD | 1 |
| with CoA & hypoplastic left ventricle | 1 |
| with dilated right heart | 1 |
| **AVSD** | **13** |
| with mitral regurgitation | 3 |
| with DORV, right sided aortic arch, common atrium | 1 |
| with hypoplastic left ventricle & mitral valve | 1 |
| with VSD | 1 |
| with Cor-Triatriatum | 1 |
| with tricuspid stenosis | 1 |
| **PFO** | **8** |
| **Outflow Malformations** |  |
| **TGA** | **18** |
| with VSD | 7 |
| with Pulmonary stenosis | 2 |
| with CoA | 2 |
| with ASD | 2 |
| with tricuspid atresia | 2 |
| with intramural left coronary artery | 1 |
| **DORV** | **3** |
| with subaortic stenosis | 2 |
| with pulmonary stenosis, right isomerisation, dextrocardia | 1 |
| with CoA | 1 |
| **Pulmonary stenosis** | **4** |
| with ASD | 1 |
| with dilated right ventricle & tricuspid regurgitation | 1 |
| **Pumonary atresia** | **2** |
| with hypoplastic right ventricle & tricuspid valve, ASD & VSD | 1 |
| **Truncus Arteriosis** | **3** |
| with intramural left coronory artery | 1 |
| **PDA** | **12** |
| with VSD | 1 |
| with tricuspid atresia & absent pulmonary valve | 1 |
| **CoA** | **17** |
| with BAV | 5 |
| with aortic stenosis | 2 |
| with VSD | 3 |
| with ASD | 2 |
| with PDA | 1 |
| **Aortic Stenosis** | **9** |
| with BAV, Aortic & mitral regurgitation | 2 |
| with ASD | 1 |
| with CoA | 1 |
| **BAV** | **4** |
| with aortic stenosis & regurgitation | 3 |
| with aortic root dilation | 2 |
| **Other Malformations** |  |
| **HLHS** | **3** |
| **Ebsteins** | **3** |
| **Univentricular heart** | **3** |
| with tricuspid atresia | 3 |
| with pulmonary atresia, hypoplastic right ventricle, PDA | 2 |
| with PFO | 1 |
| **DILV** | **6** |
| with TGA | 3 |
| with VSD | 2 |
| with IAA (A00) | 1 |
| with pulmonary stenosis, malposition of great arteries | 2 |
| with complete congenital heart block | 1 |
| **Mitral regurgitation** | **1** |
| **Common Atrium, left atrial isomerisation, bilateral SVC** | **1** |
| **Large left ventricle, tiny right ventricle, no forward flow through tricuspid valve, single outflow trunk from right ventricle** | **1** |
| **DIRV, AV valve atresia, malposition great arteries** | **1** |
| **Hemianomalous right pulmonary artery venous connection to coronary sinus** | **1** |
| **Mitral Atresia, Single Ventricle, Total anomalous pulmonary venous connection to SVS, Abnormal systemic venous connection** | **1** |
| **Hetrotoxy syndrome, situs inversus, left sided IVC, bilateral SVC (no inominate vein), TGA, pulmonary atresia, AVSD** | **1** |
| **Parachute mitral valve, VSD, left SVC, right aortic arch, left subclavian artery from pulmonary artery** | **1** |

| **Abbreviations** |
| --- |
| VSD, Ventricular septal defect |
| ASD, Atrial septal defect |
| AVSD, Atrioventricular septal defect |
| PFO, Patent foramen ovale |
| PDA, Patent ductus arteriosus |
| IAA, Interupted aortic arch |
| CoA, Coarctation of the aorta |
| DORV, Double-outlet right ventricle |
| TGA, Transposition of the great arteries |
| BAV, Bicuspid aortic valve |
| DILV, Double-inlet left ventricle |
| DIRV, Double-inlet right ventricle |
| SVC, Superior vena cava/e |
