# Supplementary material for: Genetic Variation in VEGF Does Not Contribute Significantly to the Risk of Congenital Cardiovascular Malformation
Source: PLoS One. 2009 Mar 24;4(3):e4978. doi: 10.1371/journal.pone.0004978 (PMC2654913; doi:10.1371/journal.pone.0004978)
Supplement: Table S2 — (0.06 MB DOC) [file pone.0004978.s002.doc]

**Table S2 iPLEX PCR and Extension Primers**

| **Primer** | **Sequence (5’-3’)** |
| --- | --- |
| VEGF_rs833052_Forward | ACGTTGGATGGTAGAAAACACAGCGACTGG |
| VEGF_rs833052_Reverse | ACGTTGGATGTACCAGGCTTGAAATGACAG |
| VEGF_rs833052_ Extension | CAGAGGAAATATTTGGGGCT |
| VEGF_rs833057_Forward | ACGTTGGATGAGGCTGTGGAATCACTTAAC |
| VEGF_rs833057_Reverse | ACGTTGGATGATTATGAGGAAGGGTGGGTC |
| VEGF_rs833057_ Extension | TAACAAACCTTTCTGGGC |
| VEGF_rs1547651_Forward | ACGTTGGATGTGTACTGTGTTTGCCCACTC |
| VEGF_rs1547651_Reverse | ACGTTGGATGAGAACATCAGACTGTGCTCC |
| VEGF_rs1547651_ Extension | ggTCCTGCTACCTGTCCC |
| VEGF_rs833058_Forward | ACGTTGGATGTAAAAGCCTCTTGGAGAGGG |
| VEGF_rs833058_Reverse | ACGTTGGATGCTTCTCCCTTCCATCACTTG |
| VEGF_rs833058_ Extension | aTTAACTCAAAGAAATCTAGCACTA |
| VEGF_rs699947_Forward | ACGTTGGATGAGTCAGTCTGATTATCCACC |
| VEGF_rs699947_Reverse | ACGTTGGATGTTCCCATTCTCAGTCCATGC |
| VEGF_rs699947_ Extension | CTGATTATCCACCCAGATC |
| VEGF_rs2010963_Forward | ACGTTGGATGAGAAGTCGAGGAAGAGAGAG |
| VEGF_rs2010963_Reverse | ACGTTGGATGTCCGGCGGTCACCCCCAAAA |
| VEGF_rs2010963_ Extension | gggaGTGCGAGCAGCGAAAG |
| VEGF_rs2146323_Forward | ACGTTGGATGCTTCAAACACAGTAGGAGGG |
| VEGF_rs2146323_Reverse | ACGTTGGATGATGCCACTCTTTGGAGCTTC |
| VEGF_rs2146323_ Extension | ggCTTACGTTAGATTTTGGAAGGA |
| VEGF_rs3025000_Forward | ACGTTGGATGTTGTCCCATCTGGGTATGGC |
| VEGF_rs3025000_Reverse | ACGTTGGATGGGTTTGATCCGCATAATCTG |
| VEGF_rs3025000_ Extension | GCTGGCTGGGTCACTAAC |
| VEGF_rs3025033_Forward | ACGTTGGATGTTAGGGAAGTCCTTGGAGTG |
| VEGF_rs3025033_Reverse | ACGTTGGATGATCCCCTGAGCACACACAAG |
| VEGF_rs3025033_Extension | tgGTCTCCCCTCCCCCAGC |
| VEGF_rs3025035_Forward | ACGTTGGATGTATTCCCAGATACAGCCAGC |
| VEGF_rs3025035_Reverse | ACGTTGGATGGGTTTGTGTGAAGTGACCTG |
| VEGF_rs3025035_ Extension | ttgtGGGTTGGGTAAAGGTATTG |
| VEGF_rs9369421_Forward | ACGTTGGATGTACTGCCAATGTCTGGTGTG |
| VEGF_rs9369421_Reverse | ACGTTGGATGTCTCTGAAACAGGCACCATG |
| VEGF_rs9369421_ Extension | GTGTGGGGAGGATGAC |
| VEGF_rs879825_Forward | ACGTTGGATGAGGGTTGCCAGACAAAGTAC |
| VEGF_rs879825_Reverse | ACGTTGGATGATAAAAACACGAGGTGCCCC |
| VEGF_rs879825_ Extension | gggACGGGATGCCCAGTTAAAT |
| VEGF_rs1358980_Forward | ACGTTGGATGGGGTAGGGTTTCCAAAATGC |
| VEGF_rs1358980_Reverse | ACGTTGGATGTCATGCTGGTTGCATGCTGT |
| VEGF_rs1358980_ Extension | tcTCTATGTGGGTCATGAAGT |
| VEGF_rs1885658_Forward | ACGTTGGATGATCACACTGCCCTGTCTTTC |
| VEGF_rs1885658_Reverse | ACGTTGGATGTGTCTCCCATATTCCACCTG |
| VEGF_rs1885658_ Extension | TCTAAGGAACCATATCTAGGTGTGA |
| VEGF_rs1885659_Forward | ACGTTGGATGTCAAGAGCTTTCAGTCCCAC |
| VEGF_rs1885659_Reverse | ACGTTGGATGGAAGAACTGGCCTAGAGTTG |
| VEGF_rs1885659_ Extension | tccgCTTCCTCTCTTGACTGAG |
| VEGF_rs10948095_Forward | ACGTTGGATGATCTCCATTTTCTCCCTCCC |
| VEGF_rs10948095_Reverse | ACGTTGGATGTTCTAAGAACCCCAGGAAGG |
| VEGF_rs10948095_ Extension | ggCTCCCCTCTTCTTTAGTCTTCTGCC |
| VEGF_rs13210960_Forward | ACGTTGGATGAGCCCTCTTACCCTATACTC |
| VEGF_rs13210960_Reverse | ACGTTGGATGAGGGGAGACAGAAAGTACAC |
| VEGF_rs13210960_ Extension | CACATTATAAAATGCATGTATTTATTA |
