# Supplementary material for: Genetic Variation in VEGF Does Not Contribute Significantly to the Risk of Congenital Cardiovascular Malformation
Source: PLoS One. 2009 Mar 24;4(3):e4978. doi: 10.1371/journal.pone.0004978 (PMC2654913; doi:10.1371/journal.pone.0004978)
Supplement: Table S3 — (0.04 MB DOC) [file pone.0004978.s003.doc]

**Table S3 VEGF PCR/Sequencing Primers**

| **Primer** | **Sequence (5’-3’)** |
| --- | --- |
| VEGF_1Forward | GTCCTGTTCGACTCAGAAGA |
| VEGF_1Reverse | GAACCTCGTGGTGCCCAGCT |
| VEGF_2Forward | GACTTGCCTGATTCGGAAGC |
| VEGF_2Reverse | GGGCTGGGAATGAAGCATCT |
| VEGF_3Forward | GTGCTGAGTGGCAGGAGCCC |
| VEGF_3Reverse | CAACAGAGGTAGCCAAGAGC |
| VEGF_4Forward | AGAGCCATCGAGTGCTTGCT |
| VEGF_4Reverse | CAGGGTTACAGGGAATGGCA |
| VEGF_5Forward | TGCAGCTGCGGACATGTTAG |
| VEGF_5Reverse | CAGGCTGGACCAGTGCCTGC |
| VEGF_6Forward | TATGGTGCCGGAGGCTGCAG |
| VEGF_6Reverse | AGTGCTCTGCGCAGAGTCTC |
| VEGF_7Forward | GCCAGGCTTCACTGAGCGTC |
| VEGF_7Reverse | AGCAGGTCACTCACTTTGCC |
| VEGF_8Forward | GAGATTGCTCTACTTCCCCA |
| VEGF_8Reverse | CGAGCGCCGAGTCGCCACTG |
| VEGF_9Forward | GCCGCGAGAAGTGCTAGCTC |
| VEGF_9Reverse | CTGCACCTAAGACGACAGAG |
